# Domestic horses (*Equus caballus*) discriminate between negative and positive human nonverbal vocalisations

**DOI:** 10.1038/s41598-018-30777-z

**Published:** 2018-08-29

**Authors:** Amy Victoria Smith, Leanne Proops, Kate Grounds, Jennifer Wathan, Sophie K Scott, Karen McComb

**Affiliations:** 1 Mammal Vocal Communication and Cognition Research Group, School of Psychology, University of Sussex, Brighton, BN1 9QH UK; 2 Centre for Comparative and Evolutionary Psychology, Department of Psychology, University of Portsmouth, Portsmouth, PO1 2DY UK; 3 Speech Communication Laboratory, Institute of Cognitive Neuroscience, University College London, London, WC1N 3AR UK

## Abstract

The ability to discriminate between emotion in vocal signals is highly adaptive in social species. It may also be adaptive for domestic species to distinguish such signals in humans. Here we present a playback study investigating whether horses spontaneously respond in a functionally relevant way towards positive and negative emotion in human nonverbal vocalisations. We presented horses with positively- and negatively-valenced human vocalisations (laughter and growling, respectively) in the absence of all other emotional cues. Horses were found to adopt a freeze posture for significantly longer immediately after hearing negative versus positive human vocalisations, suggesting that negative voices promote vigilance behaviours and may therefore be perceived as more threatening. In support of this interpretation, horses held their ears forwards for longer and performed fewer ear movements in response to negative voices, which further suggest increased vigilance. In addition, horses showed a right-ear/left-hemisphere bias when attending to positive compared with negative voices, suggesting that horses perceive laughter as more positive than growling. These findings raise interesting questions about the potential for universal discrimination of vocal affect and the role of lifetime learning versus other factors in interspecific communication.

## Introduction

The production and discrimination of emotional signals is a highly significant component of social living in mammals, as this allows for the efficient transmission of social intentions and the sharing of environmental information^1,2^. Emotion and arousal can be encoded through various acoustic features during vocal production, including the fundamental frequency and its harmonics (which determine pitch), as well as formant frequencies (determining timbre) and amplitude (perceived as loudness), thus providing a complex and multifaceted signal^1,3^. Vocalisations can also encode information on the signaller’s age, gender, and identity^4^, and so can provide receivers with a wide range of information. Considering the importance of vocalisations in promoting effective communication, species with frequent human contact may benefit from attending to the social and emotional information within human vocalisations, and from adjusting their social interactions with humans accordingly.

The emotional cues contained within vocalisations have the potential to follow similar acoustic rules across human and nonhuman species (the motivational-structural rules hypothesis^5^; sound symbolism^6^). Harsh, low-frequency sounds are typically used in threatening contexts whilst higher, relatively pure-tone frequencies tend to be used in appeasement or affiliative contexts^5,7^. It is suggested that these variations in acoustic structure may also be used ritualistically to mimic differences in body size and therefore alter the perceived level of threat posed by the signaller^1,8^. Lower fundamental frequencies can generate the impression of a larger body size^5^, along with lower vocal tract resonances (formants), which suggest a longer vocal tract^6^. Moreover, emotional states can directly alter the sound produced in the larynx due to changes in the rate of respiration and in the tension of the vocal folds^1^. The facial expression associated with the affective state can also influence the sound, through its effect on mouth shape and consequent filtering^1,3,9,10^. Such fundamental similarities in the form of affective vocalisations across species may facilitate interspecific communication of emotion.

For domestic animals it would be particularly advantageous to discriminate between positive and negative affect in humans. Numerous studies have demonstrated that domestic dogs are able to discriminate the emotional content of human voices in a range of contexts. Using a cross-modal emotion perception paradigm, dogs were found to associate positive and negative human emotional vocalisations with the corresponding facial expressions^11^ (but see^12^). In addition dogs are more likely to avoid contexts involving a scolding human versus dehumanised vocalisations and control conditions regardless of the signaller’s gender^13^ and to obey pointing commands more successfully when issued in a high-pitched, friendly voice compared with a low-pitched, imperative voice^14^. Furthermore, neurological fMRI research reveals different patterns of neural activity in dogs when hearing high-pitched praise versus neutral voices^15^. However, very few studies have investigated such abilities in other domestic species, and further, recent empirical evidence has suggested that horses do not differentiate between a harsh and a soothing voice when being trained to cross a novel bridge^16^. The authors suggest that the horses may not have attended to the voices due to the potentially more salient training cue of pressure release on the halter that was used as an additional signal in the experimental paradigm. New paradigms are therefore needed to fully explore horses’ abilities to discern emotionally relevant cues in human vocalisations.

Despite the lack of evidence to date, horses are potentially good candidates for having abilities relevant to discriminating between vocally expressed emotions in humans. Horses are sensitive to cues of affective state in conspecific vocalisations^17^ (see also^18^) and therefore may be predisposed to attend to emotional cues embedded in vocalisations generally. They have also been shown to discriminate socially relevant cues in human voices, such as voice identity characteristics during individual recognition^19^. Moreover, horses can distinguish human emotional states through other modalities such as through facial expression^20^, and are sensitive to changes in human anxiety levels^21^. As humans use their voices extensively during direct interaction with horses in riding, training, and groundwork it is likely that horses would also benefit from discriminating between different emotions expressed in human voices, as this would allow them to better predict the consequences of their interactions with humans.

In this study we used playback of auditory stimuli to investigate whether or not horses respond differently to positive and negative emotions displayed in human vocalisations. We presented horses with male or female human nonverbal vocalisations characterised as either happy (laughter) or angry (growling). Each horse was presented with one positive and one negative vocalisation of either a male or female human, in tests separated by at least one week. We predicted that there would be more negative responses towards negative vocalisations (more vigilance and freeze behaviour, avoidance, displacement behaviours, and left ear/right hemisphere biases) and more positive responses towards positive vocalisations (more approach behaviour and right ear/left hemisphere biases). In addition we predicted that horses would respond more negatively towards male stimuli versus female stimuli due to the relatively lower pitch and formant frequencies that are characteristic of male voices^10^.

**Figure 1:**
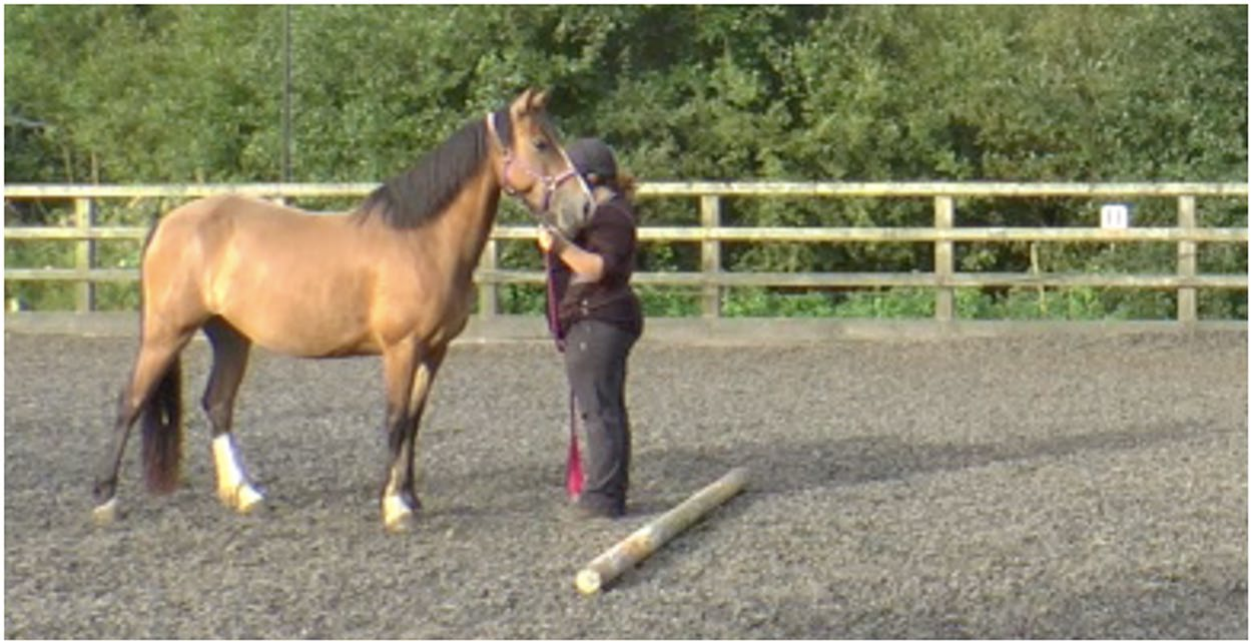
The test position: Horse is held perpendicular to the speaker that is hidden 15 m away amongst vegetation (beyond right of photo).

Thirty-two horses took part in two trials each, one of which presented a negative and one a positive human vocalisation. Each horse received either male or female stimuli but not both. Trials were separated by at least one week (*M* = 18.57 days, *SD* = 8.26, max = 29 days). Emotions and stimuli were counterbalanced equally between horses and across trials. Stimuli were played through a MIPRO MA707 battery powered speaker connected to a Macbook Pro, which were placed 7 m outside a fenced riding arena and concealed within wooded vegetation. Horses were held parallel to the speaker 8 m from the fence (a total of 15 m from the speaker) at a line marked with a familiar jump pole (Fig. 1). During trials the horse was initially held for 2 min in the test position (perpendicular to the jump pole and directly facing the hidden speaker) to get used to the experimental setup. Following this lag period the stimulus was played once and then repeated after 10 s of silence. After the stimulus presentation the horse was held in the test position for a final 2 min. See Method for full details.

## Results

### Behavioural responses

**Figure 2:**
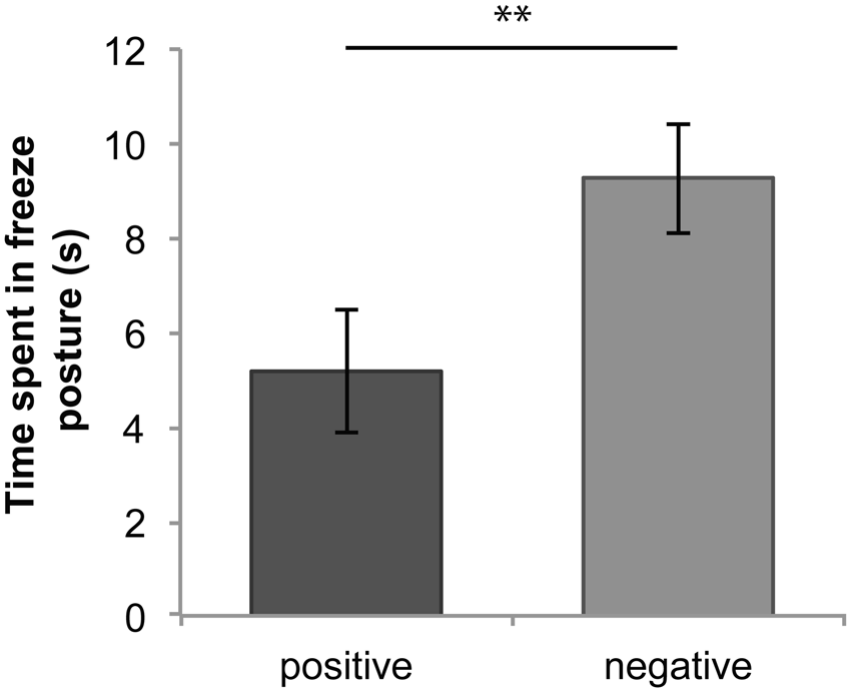
Mean time spent in freeze posture by emotion (±1 *SEM*) **p < 0.01.

On average, horses adopted a freeze posture for significantly longer after hearing negative (*M* = 9.27, *SEM* = 1.17) compared with positive (*M* = 5.18, *SEM* = 1.33) vocalisations, *F*(1,52) = 8.32, *p* = 0.006 (Fig. 2). The stimulus gender did not have a significant effect on time spent in freeze behaviour, *F*(1,52) = 3.07, *p* = 0.086, and there was no significant interaction between stimulus emotion and gender, *F*(1,52) = 1.09, *p* = 0.30.

**Figure 3:**
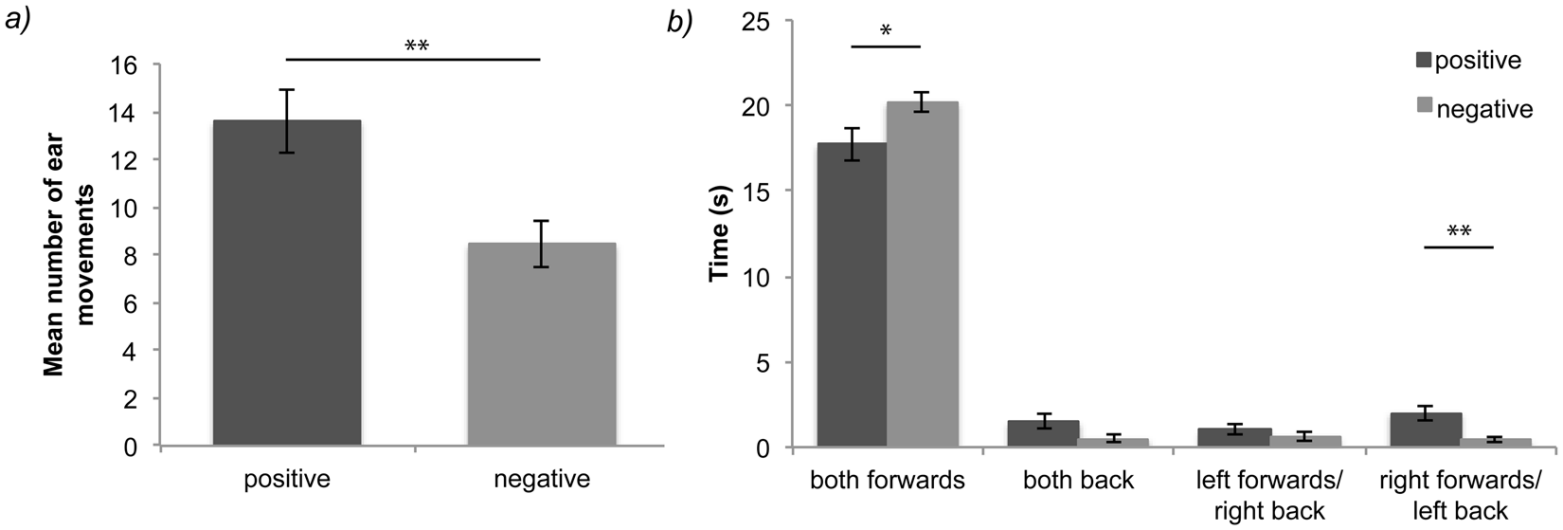
(**a**) Mean number of ear movements during trial by emotion (±1 *SEM*); (**b**) mean time spent displaying patterns of ear behaviour, *p < 0.05, **p < 0.01.

Horses performed a significantly higher number of ear movements overall towards positive (*M* = 13.61, *SEM* = 1.33) compared with negative (*M* = 8.46, *SEM* = 0.97) vocalisations, *F*(1,52) = 10.52, *p* = 0.002 (Fig. 3a). Here stimulus gender did not have a significant effect on the number of ear movements, *F*(1,52) = 0.28, *p* = 0.60, and there was no significant interaction between stimulus gender and emotion on the number of ear movements, *F*(1,52) = 0.20, *p* = 0.65.

There was a significant interaction between emotion and ear behaviour, *F*(3,208) = 7.34, *p* < 0.001. Horses held both ears forwards for significantly longer towards negative (*M* = 20.22, *SEM* = 0.58) compared with positive (*M* = 17.73, *SEM* = 0.94) vocalisations, *t*(208) = 2.61, *p* = 0.04. Further, horses had a significant preference for holding their right ear forwards and left ear back towards positive over negative vocalisations, *t*(208) = 3.60, *p* = 0.004 (positive *M* = 2.02, *SEM* = 0.46; negative *M* = 0.43, *SEM* = 0.14). There were no significant differences in time spent with both ears backwards, *t*(208) = 1.94, *p* = 0.22 (positive *M* = 1.49, *SEM* = 0.39; negative *M* = 0.50, *SEM* = 0.22), nor with left ear forwards/right ear back, *t*(208) = 1.34, *p* = 0.72 (positive *M* = 0.99, *SEM* = 0.29; negative *M* = 0.64, *SEM* = 0.27) (Fig. 3b). Stimulus gender did not have a significant interaction with ear behaviour, *F*(3,208) = 2.20, *p* = 0.089, and there was no significant interaction between stimulus emotion, gender, and ear behaviour, *F*(4,208) = 1.36, *p* = 0.25.

Too few horses engaged in the additional behaviours to allow statistical analysis: in response to positive stimuli, approach *n* = 3, avoid *n* = 6, lick and chew *n* = 3, head bob *n* = 0, head shake *n* = 1, scratch *n* = 1, paw ground *n* = 2. In response to negative stimuli, approach *n* = 4, avoid *n* = 2, lick and chew *n* = 3, head bob *n* = 0, head shake *n* = 0, scratch *n* = 0, and paw ground *n* = 1.

### EquiFACS results

None of the measured EquiFACs action units had significant relationships with emotion (full exploratory analyses in Table 1). Where 5 or fewer horses performed the action, statistical tests were not performed (AU10 – upper lip raiser; AU18 – lip pucker; AU12 – lip corner puller; AD160 – lower lip relax; AU24 – lip presser).

**Table 1:** EquiFACS action unit codes, descriptives, and exploratory GLMM results.

Action Unit	Descriptor	Emotion	*Mean*	*SD*	*F*	Exact Sig. (2-tailed)
AU101 time	Inner brow raiser	Positive	14.01	6.68	1.36	0.25
		Negative	15.79	6.69		
AU145 count	Blink	Positive	4.18	2.60	2.79	0.10
		Negative	3.14	2.85		
AU47 count	Half blink	Positive	3.57	2.12	0.10	0.76
		Negative	3.36	3.21		
AU5 time	Upper lid raiser	Positive	3.37	5.46	0.44	0.51
		Negative	4.29	6.47		
AD1 time	Increased eye whites	Positive	3.76	5.42	0.06	0.81
		Negative	3.47	4.67		
AU113 count	Sharp lip puller	Positive	0.43	1.03	0.30	0.59
		Negative	0.32	0.55		
AU16 count	Lower lip depressor	Positive	0.54	1.40	0.08	0.78
		Negative	0.64	1.50		
AU17 count	Chin raiser	Positive	1.46	2.19	0.009	0.91
		Negative	1.43	2.12		
AUH13 count	Nostril lift	Positive	0.79	1.10	1.41	0.24
		Negative	1.14	1.51		
AD113 count	Blow	Positive	0.57	1.42	1.73	0.19
		Negative	0.29	0.71		

## Discussion

Horses adopted a freeze posture for significantly longer immediately after hearing negative versus positive human vocalisations, a posture which is characterised by forward attention and a lack of movement and is often given in response to an environmental threat^22^. Horses therefore appear to discriminate behaviourally between different types of human vocalisation, in ways that suggest they perceive negative human voices as more threatening than positive voices. In support of this interpretation, horses held their ears forwards for significantly longer, indicating increased vigilance, and performed significantly fewer ear movements in response to the negative vocalisations.

The freeze response forms part of the ‘fight, flight, or freeze’ reaction to a perceived threat^22^. The individual increases vigilance towards an object of interest by orienting the head, eyes, and ears intently towards the stimulus and reducing muscle movement, which reduces the risk of detection and readies the muscles for a fight or flight response^22,23^. In horses this posture is in stark contrast to a relaxed state in which the ears are laterally placed and the ears and head are moving frequently^24,25^. Individuals typically freeze in response to a distant and relatively mild threat, whilst closer and more extreme threats may provoke vocalisations, direct avoidance, and attack behaviours^26^. The freeze response therefore appears to be an appropriate reaction in the present paradigm where the stimulus is mildly aversive and distant, i.e. comfortably outside the horse’s flight zone^27^.

In addition to freeze behaviour, horses displayed some evidence of a right auditory lateralisation towards the positive vocal signals. In many species, a right-ear bias indicates that signals are preferentially processed in the left brain hemisphere and are generally associated with the perception of familiar or positive stimuli^28,29^. Auditory laterality in horses has not been studied extensively, although, research suggests that incoming signals are processed primarily in the contralateral brain hemisphere^30^. Auditory laterality in horses has not been established in relation to emotional contexts, however horses show auditory laterality in social situations^30^ and demonstrate both gaze and limb preferences in emotional situations^20,31–33^. Lateralised ear behaviour may therefore also be interesting in an emotional context. Although the right-ear lateralisation we observed could indicate a lateralized discrimination between human vocalisations, it is notable that throughout the trials horses displayed relatively little lateralised ear behaviour. Their preference to hold both ears forwards during trials, in order to attend to the stimulus, could have masked any potential left-ear preferences that might have been expected in the case of reaction to negative voice cues. The lateralised ear behaviour in the present paradigm should therefore be interpreted cautiously. Similarly, the strong freeze response may have prevented any differences in approach, avoidance, and displacement behaviours from emerging.

The ability of nonhuman species to discriminate between human vocalisations raises interesting questions about the potential universality of emotion discrimination through auditory signals and determining the particular acoustic parameters that give rise to this discrimination would be a useful avenue for future research. For example, negative emotional arousal is expressed through harsh, low-frequency tones across a wide range of species, and so these cues may be readily responded to even in the vocalisations of other species, without explicit prior experience of these species^1,5,6^. Future experiments could usefully explore the extent to which this response generalises to the negative sounds of a range of other species, comparing such responses to general reactions to low frequency sounds produced by inanimate objects in the environment (e.g. machinery noises). Further studies could also investigate the contribution of innate factors and lifetime experience, as well as the role of recognition versus discrimination, in driving these processes.

Interestingly, horses did not discriminate behaviourally between male and female voices in the paradigm we used. We had predicted that horses would respond more negatively to male voices, and specifically negative male voices, due to their having relatively lower fundamental and formant frequencies than female voices^10^. However, there is similar evidence that dogs do not discriminate the sex of a human signaller when hearing emotional vocalisations^14^ despite having the ability to discriminate gender in human voices^34^. It is therefore possible that emotional cues are more salient than gender cues in such paradigms and so are responded to preferentially.

In the present study, the use of laughter and growling vocalisations as representative of positive and negative human emotions introduced a slight difference in stimulus length, with an average positive vocalisation of 1.78 seconds and average negative vocalisation of 1.14 seconds. Human laughter is characterised by voiced pulses interspersed with pauses^35^ compared with the lack of pauses in growling vocalisations, and these acoustic characteristics were adopted naturally by the actors during the recording of the stimuli used (previously validated in Sauter *et al*.^36^). The variance in stimulus length is therefore considered to represent naturalistic and distinctive differences between the two emotional expressions and so was considered appropriate. Further, if stimulus length had an influence on behaviour times, one would expect shorter freeze and binocular looking times to the shorter, negative vocalisations, whilst the opposite was in fact observed here.

These results complement the current body of research on dogs’ abilities to discriminate human vocal emotions^11,13^, extending this work to another key domesticated species. While previous research by Heleski *et al*. had suggested that horses do not discriminate between harsh or soothing human voices^16^, our results present a different picture. The difference in experimental paradigm may conceivably have led to these contrasting results. The use of a training paradigm and additional cues, as in Heleski *et al*.^16^, may add confounding variables that could mask potential differences. Spontaneous discrimination paradigms, such as that used in the present study, may be best placed to detect subtle differences in behavioural responses.

## Conclusions

In our experiments, horses discriminated between human nonverbal emotional signals, exhibiting increased vigilance, including freeze behaviour, towards negative versus positive human emotional vocalisations, and displaying a right ear (left hemisphere) bias for positive versus negative vocalisations. These findings add to previous literature on dogs’ abilities to discriminate emotion from human voices, extending our knowledge of interspecific communication and raising interesting questions about the extent to which vocal signals of emotion are discriminated universally or learnt through experience.

## Method

### Subjects

32 horses were recruited from two riding schools in East Sussex, U.K., between August 2015 and March 2016. Horses who were distracted for more than 15 seconds during the trial were excluded (*n* = 4), leaving 28 horses in the final analyses (17 geldings, 11 mares; age range 7–22 years, *M* = 15.71, *SD* = 4.80).

### Stimuli

**Figure 4:**
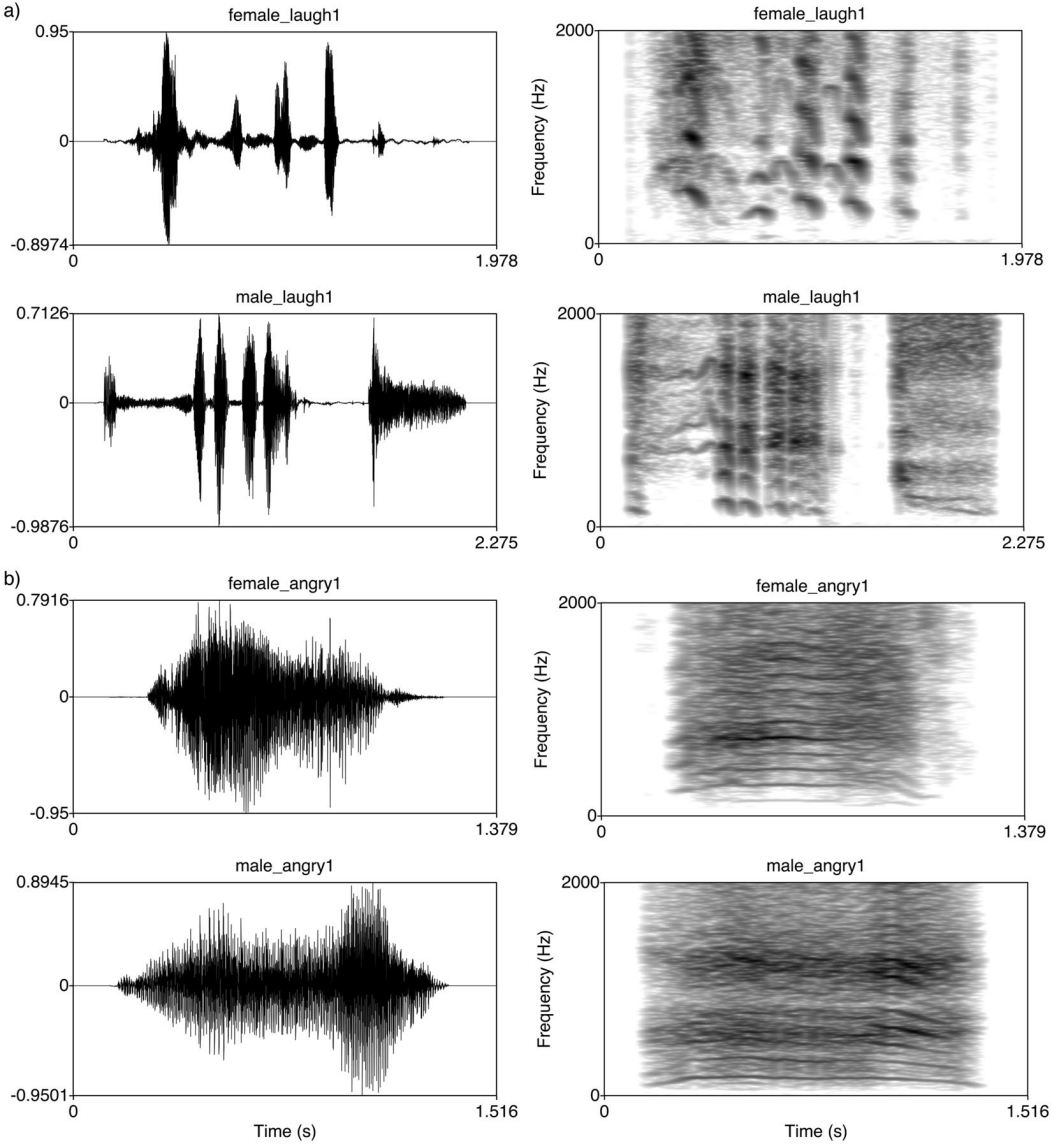
Example spectrograms and waveforms of (**a**) positive (laughter) and (**b**) negative (growling) vocalisations; top rows = female, bottom rows = male.

Eight human nonverbal emotional vocalisations were used as exemplars: four positive vocalisations represented by laughter (two male, two female) and four negative vocalisations represented by growling (two male, two female) (Fig. 4). Sound files were obtained from a previously validated set of nonverbal affective vocalisations recorded in an anechoic chamber^36^. Stimuli were reconfigured for the current experiment using Praat v.5.2.21 and Audacity v.2.1.0. Specifically, vocal sequences of approximately 1–2 s were extracted from sound files (range for positive vocalisations: 1.43–2.14 s, *M* = 1.78; range for negative vocalisations: 0.97–1.19 s, *M* = 1.14). The slight differences in vocalisation length reflect ecologically valid vocalisation times. Each sound file contained one vocalisation, which was repeated after 10 s. Each sound file was therefore approximately 13 seconds long, with some variation depending on the length of the stimulus. Stereo files were converted to mono and stimuli were normalised to either 95% or 99% peak intensity (depending on original sound pressure level) and broadcast at levels of 100 dB at 1 m from the source.

### Procedure

Trials were conducted in a familiar outdoor riding arena. Two cameras (wide-angled Panasonic HC-X920) were positioned on tripods 10 m away and 3.5 m to the right of the jump pole to obtain a ¾ view of the horse’s face. Camera one captured whole body behaviour and camera two captured detailed facial behaviour. Throughout the trial the handler (experimenter 1) stood beside the horse’s head facing away from the speaker, avoided interacting with the horse, and wore small earpiece headphones attached to an MP3 player (through which they listened to music) so they could not hear the playbacks and remained blind to the stimuli. Horses were held on a 1 m lead rope and gently encouraged to keep their head facing forwards. If the horse moved out of the test position the handler led them back into position. Experimenter 2 operated the speaker and the cameras, and kept one camera trained on the horse’s face throughout the trial to capture detailed facial and ear behaviour.

### Behavioural and statistical analyses

Behaviours measured were: ear position (time spent with both ears forwards, both ears back, left ear forwards/right ear back, and right ear forwards/left ear back); number of ear movements; time spent performing approach and avoidance behaviours (defined as any bodily or leg movement towards or away from the stimulus source respectively); time spent in freeze behaviour (attentive and oriented towards the stimulus source, both ears held forwards, and a lack of head, neck, or ear movement apart from blinking and slight nostril movements); and frequencies of displacement behaviours during the test (lick and chew behaviour, head bobbing, head shaking, and pawing the ground). Additionally we coded facial responses using a subset of EquiFACS action units^37^ to investigate potential differences in detailed facial behaviour (see results section for details of the action units measured; see supplementary material for data files). All behaviour was coded between the onset of the first stimulus and 10 s after the second stimulus ended; trial length therefore varied slightly depending on the length of the vocalisation (length of trial (s): *M* = 23.16, *SD* = 0.76, min = 21.88, max = 24.60). Lower face movements were not coded whilst horses were walking due to this motion potentially causing additional movements. One horse was excluded from the AU101 (inner brow raiser) analysis as their mane covered their brow during the trial. Videos were blind-coded using SportsCode Gamebreaker Plus v.10.1 software. Twelve videos (21.5%) were double-coded by certified EquiFACS coders, showing good reliability in EquiFACs codes with an ICC of ≥0.79 (*M* = 0.90, *SD* = 0.08) and in behaviour codes with an ICC of ≥0.91 (*M* = 0.96, *SD* = 0.03) (two-way mixed single-measures ICCs using absolute agreement). Statistical analyses were performed using Excel and SPSS 22.0 on a MacBook Pro.

Differences in freeze behaviour and number of ear movements were analysed using generalized linear mixed models (GLMMs) with presentation round as a repeated factor (round 1 and round 2; repeated covariance type: scaled identity), stimulus emotion (positive/negative) and stimulus gender (male/female) as fixed factors, and subject as a random factor (including intercept). Differences in ear behaviour were tested using the same GLMM model parameters with ‘ear behaviour’ (both forwards, both back, left forwards/right back, and right forwards/left back) as an additional variable. Post-hoc comparisons were corrected using the Bonferroni statistic. Exploratory GLMMs, model parameters as above, were run to investigate differences in EquiFACS action units using stimulus emotion as the fixed factor. Too few instances of approach, avoidance, and displacement occurred to allow statistical analysis.

### Ethical statement

This research follows Association for the Study of Animal Behaviour Guidelines for the Use of Animals **(**Animal Behaviour, 2006, 71, 245–253) and was approved by the University of Sussex Ethical Review Committee (ERC), reference number: Non-ASPA 3–January 14. Informed consent was gained from stable owners.

## Electronic supplementary material

Dataset 1

## Data Availability

The datasets generated during the current study are available in the supplementary material.
